# Addressing Mass Transport Limitation and Gas Crossover Behavior in Zero-Gap Porous Separator Configurations for Alkaline Water Electrolysis

**DOI:** 10.1021/acsaem.5c02750

**Published:** 2025-12-01

**Authors:** Adrian Hartert, Benedikt Böhm, Manuel Hegelheimer, Simon Thiele, Anna T.S. Freiberg

**Affiliations:** 1 Helmholtz-Institute Erlangen-Nürnberg for Renewable Energy (IET-2), Forschungszentrum Jülich, Cauerstr. 1, Erlangen 91058, Germany; 2 Department of Chemical and Biological Engineering, 9171 Friedrich-Alexander-Universität Erlangen-Nürnberg, Cauerstr. 1, Erlangen 91058, Germany

**Keywords:** liquid alkaline water electrolysis, gas bubble accumulation, gas crossover, mass transport limitation, ohmic resistance in AWE

## Abstract

Traditional alkaline water electrolysis (AWE) is an established technology for green hydrogen production but suffers from high overpotentials and balance of plant costs. Efforts to allow high current densities have included a zero-gap design but so far neglected the parallel advancements in related membrane electrolysis technologies. This study therefore employs a 5 cm^2^ zero-gap AWE setup with a porous diaphragm, nanostructured electrodes, and mild electrolyte concentrations of 1 M KOH. Initially, the system is bound to low current densities by mass transport limitation. By variation of the compression and comparison of different porous transport layers (PTLs), a structure–performance relationship is developed. The gas purity and attained hydrogen flux at the cathode are analyzed simultaneously. Configurations with low overpotentials are identified to commonly bear high crossover rates so that optimization toward efficient hydrogen production can be achieved only when electrochemical and gas crossover analyses are paired. A potential solution is found when the path of the liquid electrolyte within the cell is modified. By forcing convective transport through the PTL, the gas bubbles are removed efficiently. Ultimately, the system is able to reach current densities above 2.5 A cm^–2^ at 2.3 V while keeping adequate gas purity.

## Graphical abstract

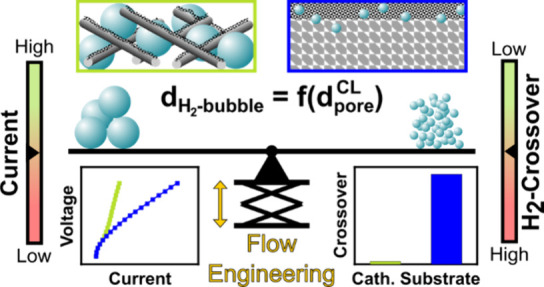

## Introduction

Green hydrogen is experiencing a remarkable momentum around the world in its role not only as a potential energy carrier^1^ but also as a key chemical in enabling the future transformation of several industrial processes (e.g., ammonia and steel production) toward decarbonization.^2^

Alkaline water electrolysis (AWE) is the most mature technology to produce said green hydrogen at a large scale today, if renewable energy is employed in the process. A conventional alkaline water electrolyzer consists of two electrodes (usually nickel-based) in a liquid electrolyte and a diaphragm at the center for gas separation. However, one drawback of AWE is the low operating current density, caused by high ohmic losses due to the relatively large effective distance between the electrodes in the millimeter range,^3–5^ which is necessary to avoid gas crossover.^6,7^ To improve ion conduction^8^ and decrease gas solubility,^3,9^ traditional alkaline electrolyzers typically employ highly concentrated (30%_wt_ ≈ 7 M^10^) KOH as a liquid electrolyte, which accelerates corrosion of materials^11^ and leads to high costs for the balance of plant components.^12^ One important aspect for AWE is the increase in ohmic resistance upon current density increase, which is seen to be caused by imperfect bubble removal^4,5^ and can be partially eliminated by flow engineering.^13,14^

The second industry ready technology for water splitting is proton exchange membrane water electrolysis (PEMWE), where the use of a solid polymer electrolyte (membrane) allows for an efficient zero-gap design. The additional use of nanostructured porous electrodes made from high surface area materials results in significantly improved efficiencies and dynamic operation behavior of this technology over those of its predecessor. The major disadvantage of this concept is the mandatory use of platinum group metals (PGMs) as catalyst materials because of the harsh acidic environment.

Anion exchange membrane water electrolysis (AEMWE) is an upcoming technology, which both promises the high efficiencies of a zero-gap design (adapted from PEMWE) and the usage of abundant and often non-noble materials while allowing moderately concentrated electrolytes (typically 1 M ≈ 5%_wt_ KOH^10^). Despite potentially presenting a lot of advantages, this technology is still in its infancy, with one bottleneck being the ionic conductivity and stability of anion exchange polymers.^15^

Several studies employing zero-gap AWE setups have been performed in an effort to reduce the known losses of this traditional technology. However, the majority of this research has adopted the rudimental electrodes and/or employed highly concentrated electrolyte solutions from traditional AWE, as has been comprehensively illuminated by de Groot and Vreman^5^ and Henkensmeier et al.^16^ Additionally, true zero-gap configurations of AWE cells have been shown to suffer from increased gas crossover.^17^

In a recent study by Demnitz et al.,^18^ it was demonstrated that PGM-free AWE cells can reach current densities of 3.5 A cm^–2^ at less than 2.3 V by employing a catalyst-coated diaphragm. Rocha et al.^19^ showed excellent electrochemical performance by employing three-dimensional electrodes in a flowthrough design that allows operation at 2 A cm^–2^ at less than 2 V. However, both studies employed high electrolyte concentrations of 30%_wt_ and electrodes with high loadings and product gas analysis was not reported.

In this study, we employ state-of-the-art nanostructured catalyst layers, adapted from PEMWE, to enable high performance in a zero-gap AWE design with 1 M KOH as the supporting electrolyte, which allows the usage of cost-effective materials in the future. This study aims to help understand the unique structure–performance relationship regarding mass transport limitation issues when dealing with porous separators instead of ion conductive polymer materials. To not revert the original function of the gap, an often-neglected gas stream analysis was incorporated to monitor the gas crossover through the porous separator. Finally, we propose a modified liquid flux configuration with a flowthrough electrode, which enabled current densities of >2.5 A cm^–2^ at 2.3 V.

## Experimental Section

### Electrode Fabrication

All electrodes were fabricated by spray coating catalyst ink on top of the respective porous transport layer to form a catalyst coated substrate (CCS). The CCS approach was used rather than applying the catalyst onto the diaphragm in order to achieve samples with homogeneous loading. Both anode and cathode catalyst layers are PGM-based as structural changes of the catalyst during electrochemical analysis as, e.g., observed for Raney-nickel complicate the structure–performance relationship that we aim to understand.

For ink fabrication, all chemicals were employed as received. The catalysts chosen were IrO_*x*_ (Premion, Thermo Scientific) for the anode and Pt/C (60%_wt_ Pt on C, HiSPEC, Thermo Scientific) for the cathode. Nafion (D520, Ion Power Inc.) was used as a binder in both electrodes, whereas the amount of polymer dispersion added was chosen to result in a solid fraction of 10%_wt_ of the polymer with respect to the catalyst. By addition of a 1-propanol (>99.0%, Emplura, Merck KgaA) and DI water (Milli-Q, 18.2 MΩ cm, Merck KgaA) mixture, dilute inks with an overall solid content of 1%_wt_ were prepared. For simplicity, the solvent in the D520 dispersion was assumed to be 1-propanol only. The solvent weight ratio of the ink was 1:1 DI water and 1-propanol for anodes and 3:1 for cathodes.

The mixture was stirred overnight before being additionally dispersed by an ultrasonic horn (UP200 St, Hielscher Ultrasonics GmbH) at 40 W three times for 20 min while being cooled in an ice bath. The ink was then sprayed onto the substrates with an ultrasonic spray coater (ExactaCoat, Sono-Tek Corporation) in a similar fashion as previously established within our group.^20^ For the anode electrodes, Ni-fiber substrates (Currento PTL Ni-60/250, Bekaert, measured thickness: ∼280 μm) were used throughout the study. For cathodes, the same Ni-fiber substrate and two different carbon paper PTLs were used (H23C2 and H24C5, Freudenberg Performance Materials SE & Co. KG, measured thickness: ∼230 μm). The catalyst loading was determined by weighing the substrates before and after the spraying process, whereas loadings of 2.0 ± 0.1 mg_IrOx_ cm^–2^ for anodes and 0.5 ± 0.05 mg_Pt_ cm^–2^ for cathodes were utilized. Afterward, the electrodes were tempered at 150 °C for 15 min in an oven (VD056–230 V, Binder GmbH) to anneal the Nafion. It should be noted that Nafion was used as a binder due to its good ink and catalyst layer stabilization properties. The acidic headgroup was completely neutralized upon contact with KOH due to the condensation reaction, resulting in a simple Thermoplast as binder.

### Cell Assembly

Before assembling a cell, the porous separator (Zirfon, UTP 220, Agfa-Gevaert Group) was cut in a 24 cm^2^ square and immersed in 1 M KOH (from KOH pellets, Normapur, VWR International LLC, 10 ppm of Fe) for 24 h.

Within the cell, the frame of Zirfon around the active area was compressed as much as possible to prevent leakage. Originating at 220 μm, the resulting thickness was measured (S112XB, Mitutoyo Corp.) to be 150 μm, and a 145 μm PTFE gasket (Fiberflon) was installed around the separator. The 5 cm^2^ electrodes on each side of the cell were also surrounded by gaskets to control the compression within the active area. In total, the compressible thickness therewith results in the sum of the electrodes and the compressible portion of the Zirfon (70 μm). The compression is defined as the difference between the electrode gasket thickness and the compressible thickness normalized to the compressible thickness. The gasket thicknesses on each side were chosen symmetrically.

In the spacer configuration, an ∼150 μm stainless steel fiber sinter (Currento PTL SS-74/145, Bekaert) was placed between the cathode and separator, whereas the gasket thickness around the electrode and spacer was increased by 150 μm to keep the compression constant.

The membrane-separator assembly was then placed in an in-house designed cell hardware consisting of Au-coated flow fields (anode: Monel, cathode: titanium) with a single-channel serpentine flow pattern (5 cm^2^ active area, 1.0 mm channel width, 0.8 mm land), copper current collectors, and aluminum backplates. The cell was fixed stepwise by twelve M8 screws in a cross-like pattern to a final torque of 15 N m.

For the flowthrough electrode configuration, the channels of the flow field were blocked by 3D-printed (X1-Carbon, Bambu Lab) pieces of a KOH-resistant filament (Flexfill TPE 90A, fillamentum), and the cut-out in the electrode gasket was adjusted to allow liquid electrolyte flow in the in-plane direction through the electrode.

### Test Station

After a cell was assembled, it was connected to a self-built test station, where it was fed with 1 M KOH on both half-cells. The cell temperature was controlled to be 70 °C by four heating rods. Each electrode had its own separated electrolyte loop and reservoir (PTFE tubes and bottles, 2 L each, filled with 1 L KOH), from where the electrolyte was pumped at 30 mL/min via a peristaltic pump through a heating bath for preheating before reaching the cell. The electrolyte reservoir was constantly flushed by 400 sccm N_2_ (controlled by digital mass flow controllers) to prevent any explosive atmosphere from forming, reduce the response time of the gas stream analysis, and to serve as an internal standard for the latter. The gas streams were then bubbled through two condensate traps (the second of which was actively cooled to 4 °C) for drying of the gases. Last, the current collectors of the cell were connected to a potentiostat (VMP-300 with 3 × 10 A booster, BioLogic SAS) for electrochemical characterization.

### Electrochemical Protocol for Full Cell Testing

To account for drastically different performances between the different tested cell configurations, the protocol for electrochemical testing was designed to be fully voltage controlled (except for the gas stream analysis) to ensure the same number of measurement points for each cell configuration and therefore provide comparable results. Each cell configuration was tested three times, of which the mean value was plotted and the standard deviation was represented by error bars.

The testing protocol incorporated a 1.5 h rest period at the beginning of the procedure. During this period, the cell reached a stable operating temperature, while the electrolyte was flushed with N_2_ and pumped through the cell. After that, voltages of 1.1, 1.2, and 1.3 V were applied stepwise to test for any electrical short current, which was always well below 1 mA cm^–2^. The voltage was then slowly increased to 1.8 V at a rate of 10 mV s^–1^, and then, the voltage was held for 2 h to break in the cell. Afterward, a begin of test characterization was conducted. A polarization curve was recorded between 1.4 and 2.3 V in steps of 0.05 V, with a holding time of 3 min at each voltage, of which the last 30 s were averaged to quantify the current response. This was followed by a short impedance sweep (freq.: 200 kHz to 1 Hz, at 10 mV perturbation) for ohmic resistance determination at each point. Impedance spectra were fitted with an equivalent circuit model consisting of an inductor, an ohmic resistor, and a transmission line model,^21^ where the high frequency resistance (HFR) represents the ohmic cell resistance. Three thorough impedance spectra were obtained after the polarization curve at 1.5, 1.8, and 2.3 V (holding time: 15 min, freq.: 200 kHz to 100 mHz, 10 mV perturbation). The cell was then held at 1.8 V for 15 h in an extended constant voltage measurement, followed by the end of test characterization, which consisted of a polarization curve (forward and backward scan) and the three impedance measurements, described above.

After finishing the regular testing protocol, the cell was ramped up from resting to 500 mA cm^–2^ (at 10 mA s^–1^) and was held there for about 15 min until constant gas stream signals were obtained in the mass spectrometer data. If the cell performance allowed it (*V*_cell_ < 2.3 V), the gas streams were also analyzed at 1 A cm^–2^.

### Analysis of Gas Streams

To analyze the gas streams from the anode and cathode, they were each fed into a magnetic sector mass spectrometer (MS) (Prima BT, ThermoOnix Ltd., UK). The quantification of hydrogen, water, nitrogen, and oxygen fluxes is based on the raw signals at the respective mass to charge ratio. 400 sccm N_2_ from the MFCs in the test station was used as the internal standard, and the signals were calibrated with a factor derived from a calibration gas and corrected for the pure N_2_ background signal as described in a previous work from our group.^22^ The multisample head of the MS automatically alternated between sample streams from the anode and cathode compartments every ∼4 min (32 points, 7 s each). Once stationary, the signals were averaged over the last 4 min interval for quantification. Conventionally, crossover values are reported as %_vol_ of H_2_ in O_2_ under the premise that the absolute flux of H_2_ at the anode *ṅ*_H_2__^Anode^ is very small compared to the O_2_ flux *ṅ*_O_2__^Anode^. In this study, some configurations did not fulfill this assumption, so that all crossover data are therefore presented as the actual percentage of H_2_ in the anode gas stream.

$$x_{H_{2}}^{\mathrm{Anode}}={ṅ}_{H_{2}}^{\mathrm{Anode}}/\left({ṅ}_{H_{2}}^{\mathrm{Anode}}+{ṅ}_{O_{2}}^{\mathrm{Anode}}\right)$$ 1

For the calculation of Faradaic efficiency η_H_2__^FA^, the measured hydrogen flux in the cathode compartment *ṅ*_H_2__^Cathode^ is divided by the ideally produced amount of hydrogen *ṅ*_ideal_ at the respective current density *I*.

$$\eta_{H_{2}}^{\mathrm{FA}}=\frac{{ṅ}_{H_{2}}^{\mathrm{Cathode}}}{{ṅ}_{\mathrm{ideal}}}$$ 2

with

$${ṅ}_{\mathrm{ideal}}=\frac{I}{zF}$$ 3

In eq 3, *z* = 2 for the hydrogen evolution reaction and *F* = 96,485 C mol^–1^, i.e., the Faraday’s constant.

### Imaging

FIB-SEM images were acquired using a Zeiss Crossbeam 540 FIB-SEM, whereas the area of interest was shielded with platinum to prevent unintended beam damage. Details on the procedure can be found in the work of McLaughlin et al.^23^

For top view scanning electron microscopy (Vega 3, Tescan Group, a.s.), the electrode samples were fixed on aluminum stubs by double sided carbon tape and Au-sputtered to enhance electric conductivity. In the case of the Zirfon separator, it was soaked in a water/ethanol mixture and then dipped in liquid nitrogen to make the material brittle. It was then bent with tweezers, whereas the top skin layer fractured and partially broke off. As the material is inherently nonconductive, additional aluminum tape was used to connect the Au-sputtered surface with the sample holder. An accelerating voltage of 20 kV was used with a secondary electron detector.

## Results and Discussion

### Variation in Cell Compression for Electrodes on Nickel Fiber Substrates

While measurements using Zirfon as a separator in medium concentrated lye solutions are scarce, the expected HFR of the cells can be estimated. Using literature data for the parameters dictating the pure ohmic loss through the separator as tortuosity τ, porosity ε,^16^ and pristine thickness *t* and the intrinsic conductivity κ of 1 M KOH at 70 °C,^10^ the ohmic drop across the separator should be

$$R_{\Omega }^{\mathrm{Separator}}=\frac{\tau \cdot t}{\kappa \cdot \varepsilon }=\frac{1.75\cdot 0.022\;\mathrm{cm}}{0.3554\frac{S}{\mathrm{cm}}\cdot 0.55}=197\;m\Omega \;{\mathrm{cm}}^{2}$$ 4

When designing an alkaline electrolysis cell without a gap between electrode and separator, one detrimental parameter becomes apparent, which has, to the best of our knowledge, not been published by former studies. Adjusting the cell compression in the active area can have a significant influence on the performance and gas-crossover behavior of a zero-gap electrochemical cell.^24^ Especially with a diaphragm, which is reliant on its porosity for ion conduction, compression is believed to be of utmost importance.

The Zirfon UTP 220 has a thickness of 220 μm and can be compressed to a minimum of 150 μm, which is done around the active area to seal the cell. The resulting 70 μm plus the two porous electrodes on the Ni PTL as the substrate were considered as “compressible thickness” in this study. Four different cell compressions ranging from almost no compression (1%) to 16% were investigated first (Figure 1a). The compression was adjusted using different gasket thicknesses, as shown in the scheme.

**Figure 1:**
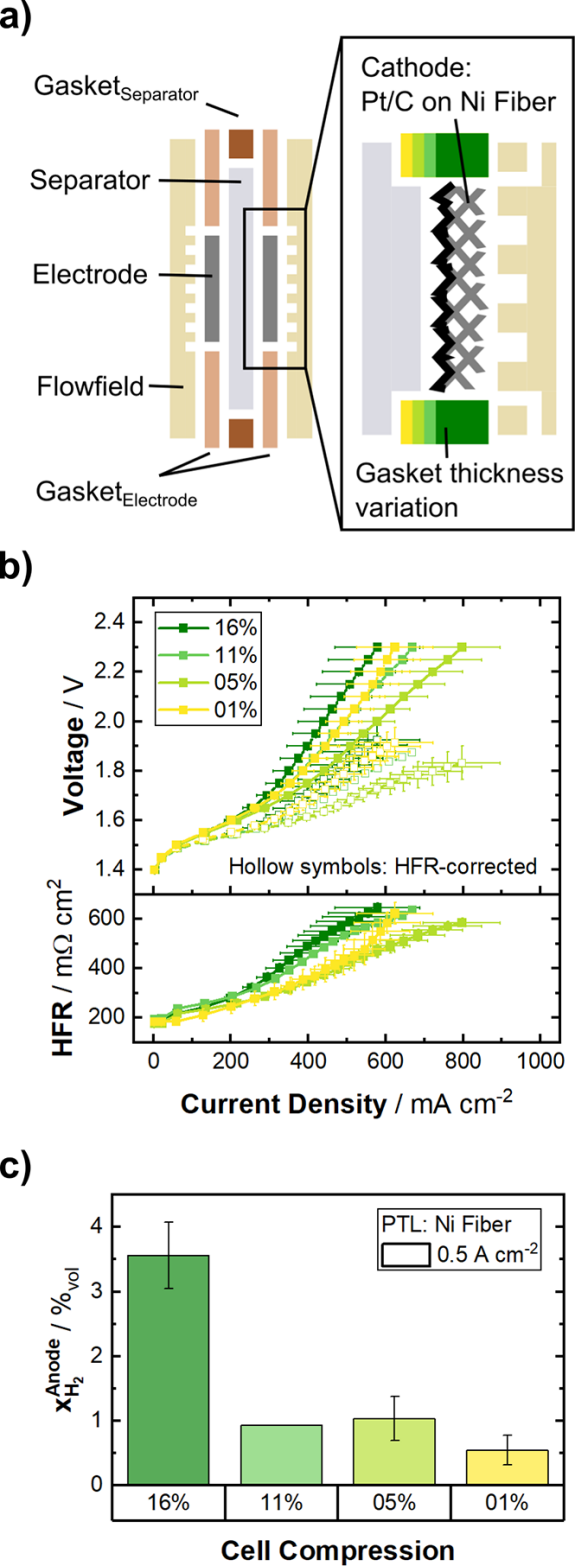
Variation in cell compression with Ni-fiber as PTL substrate on both electrodes. (a) Schematic of the cell configuration. Cell compression is adjusted by gasket thickness, which is illustrated by a gradient from dark green (highest compression) to yellow (lowest compression). (b) Potentiostatic polarization curves (top) of the considered cell compressions with their respective HFR (bottom) at 70 °C, symmetric 1 M KOH electrolyte feed and ambient pressure. The HFR-free polarization curve is shown in the top panel as hollow symbols. (c) Hydrogen crossover, quantified as H_2_ in the anode gas stream at 500 mA cm^–2^. Data are depicted as a mean value of three independent measurements with error-bars denoting the standard deviation.

Figure 1b shows the polarization curves (top) and obtained ohmic high frequency resistances (bottom) for the four tested cell compressions. The HFR at the beginning of the polarization curve fits the expected ohmic drop across the separator very well. Along the current density, the behavior of the cells can be divided into two distinct regions. Up until around 200 mA cm^–2^, all investigated compressions behave similarly with the polarization curves overlapping and the HFR increasing quasi linearly with current density.

Above this current density, the polarization curves as well as the HFR show a sharp increase, whereas the steepness and the resulting maximum current density at 2.3 V change with compression. Noteworthy is also the increase in standard deviation within the three individual measurements of each compression, resembled by the error bars (only one measurement was done at 11%). The overall performance (i.e., maximum current density) increases with decreasing cell compression for 16% (dark green), 11% (medium green), and 5% (light green), whereas the case with almost no compression (1%, yellow) does not follow this trend and shows comparably worse behavior.

Figure 1c displays the hydrogen crossover from the cathode to the anode compartment of the cell as the percentage of hydrogen present in the anode exhaust stream when the cell is operated at 500 mA cm^–2^. All four cells were below the important lower explosion limit of 4%_vol_ H_2_,^25^ and a trend of decreasing hydrogen crossover with decreasing compression is apparent.

The sharp increase in cell potential above about 200 mA cm^–2^ (Figure 1b, top) is a typical sign of a mass transport limitation onset. This means that parts of the catalyst layer (CL) are limited by reactant diffusion to or product removal from the catalytic site (e.g., by a gas bubble), which constrains the current density at a given overpotential. Denk et al.^26^ developed a model to describe the polarization characteristics of AWE in low molarity electrolytes and showed that hydroxide ion depletion at the anode (caused by the slow OH^–^ transport through the separator) can occur, leading to the emergence of a limiting current characteristic. However, for a 5%_wt_ KOH concentration, their model predicted a limiting current of more than 3 A cm^–2^ even for the thicker Zirfon Perl UTP 500 separator, i.e., more than one order of magnitude higher compared to the results in Figure 1b. Nevertheless, this current density dependent hydroxide ion depletion could explain the observed mass transport limitation, which could be reflected in both the HFR and the HFR-corrected polarization curves due to the electrolyte conductivity change within the separator and within the anode catalyst layer, respectively.

In electrolysis cells that solely rely on liquid electrolyte for ion conduction, such as the zero-gap AWE in this study, the accumulation of gas bubbles will also affect the cell’s ohmic resistance. The resistivity of a porous separator filled with liquid electrolyte depends on the porosity and tortuosity of the separator,^5,27^ as they determine the mean length of the ion pathway through the medium. Analogously, as soon as gas bubbles evolve into the liquid filled pores of the electrode, when the electrolyzer is under operation, they will occupy pore space, which is then not available for ionic conduction anymore. The bubbles therefore change the porosity and tortuosity within the pore, which causes the cell resistance to rise with increasing current density (i.e., gas evolution rate), if the bubbles are not removed effectively (Figure 1b, bottom).

Executing experiments at different lye molarities can aid in the understanding of this behavior, though one has to keep in mind the multiparameter change that is induced. The electrolyte concentration does not just change its conductivity but also vastly impacts electrode utilization, electrolyte viscosity, gas solubility, and surface tension, which largely alter bubble formation and nucleation kinetics. Experiments executed in 2 M KOH still show a distinct limiting current characteristic (data not shown). Interestingly, the polarization behavior at 1 M KOH and 2 M KOH overlaps when plotting the cell voltage over the conductivity normalized current density $\frac{i}{\kappa }$ , which is indicative of the fact that the ohmic losses^4^ caused by the electrolyte and its confinement upon bubble formation dominate the whole polarization characteristics.

By increasing the compression of the zero-gap AWE cell in the active area, the Zirfon and the electrodes get pressed together, decreasing their thickness. This changes the porosity of the components, which in result makes the cells more sensitive toward mass transport limitation, as well as increases the respective HFR. When decreasing the compression below a certain value (in this study, 5%, light green line), the ohmic cell resistance at increased current densities does not decrease further (1%, yellow line) and the current density reached at the maximum overpotential actually decreases. We ascribe this behavior to loss of contact between the Zirfon, the electrode, and the flow field, which can cause additional space for bubble accumulation (i.e., mass transport limitation) or even loss of electrical contact (i.e., common HFR). As HFR-corrected polarization curves still show this overproportional increase in potential at higher current densities (shown as hollow symbols in Figure 1b), a combination of these effects is present in our data.

The positive impact of decreasing cell compression on gas crossover (Figure 1c) is most likely a combination of multiple interconnected factors. With effectively thicker Zirfon, the mean distance between the electrodes increases, decreasing the diffusive flux between the two compartments. Additionally, the CL is pressed less into the diaphragm, which could hinder the penetration of gas bubbles into the pores of the separator.

From the first set of experiments in this study, it was found that our system is very sensitive toward compression in the active area. The phenomenon of increased potential at high current densities is mainly ascribed to mass transport limitation due to a lack of bubble removal within the electrode.

Compared to other zero-gap AWE studies, which use highly concentrated liquid electrolytes,^18,19,18,19,28,29^ this behavior is much more pronounced in our study, which can be explained by the lower specific conductivity of the employed lye. In studies that use 1 M electrolyte,^4,13,26^ very low cell efficiencies are reported, which limits the cell performance to current densities below ∼250 mA cm^–2^. In this current range, the mass transport influence is hardly observed for our configuration as well.

We show that an apparent optimum exists where compression and contact are counterbalanced. For our case, the best performing cell was found to be at 5% compression in the active area, which will be from now on taken as a reference for further comparisons in this publication.

### Carbon Paper as Cathode PTL

All tested configurations employing Ni-fiber as their cathode substrate show signs of mass transport limitation. Modern zero-gap technologies (PEMWE and AEMWE) typically apply carbon paper substrates, since they show beneficial compression properties, excellent electrical conductivity, and good permeability for reactant and product transport.^7^ The use of carbon-based materials is limited to the cathodic half-cell due to high oxidative potentials on the anode, which would lead to carbon oxidation. Consequently, we investigated two different carbon paper materials as cathode substrates for the zero-gap diaphragm configuration, of which the results are shown in Figure 2.

**Figure 2:**
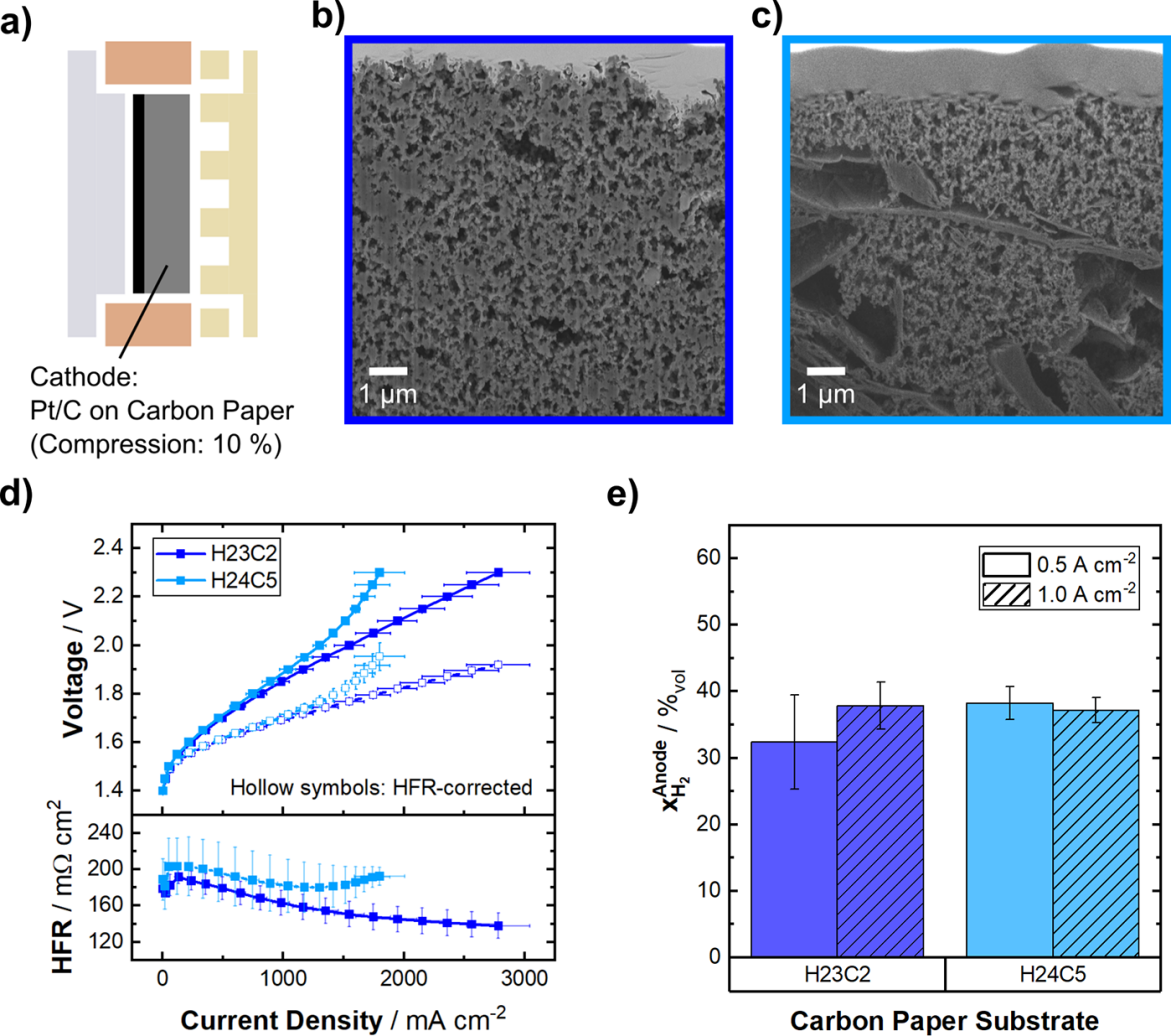
Carbon paper as a cathode PTL substrate. (a) Schematic of the cell configuration with a compression around 10%. (b, c) Cross-sectional FIB-SEM pictures of the employed carbon paper substrates, marked by frame color (H23C2: dark blue, H24C5: light blue). (d) Potentiostatic polarization curves (top) of the considered carbon paper substrates with their respective HFR (bottom) at 70 °C, symmetric 1 M KOH electrolyte feed, and ambient pressure. (e) Hydrogen crossover, quantified as H_2_ in the anode gas stream at 500 mA cm^–2^ and 1 A cm^–2^. Data depicted as mean value of three independent measurements with error-bars denoting the standard deviation.

The two substrates, originally designed for fuel cell application, have similar properties, except for the microporous layer (MPL) material, which is carbon black (with 20%_wt_ PTFE) for the H23C2 and graphitized carbon (40%_wt_ PTFE) in the case of the H24C5.^27^ The respective cross sections of the MPLs are shown in Figure 2b and c (H23C2: dark blue border, H24C5: light blue border). While the H23C2 shows a highly porous, homogeneous structure, the architecture of the H24C5 exhibits flake-like formations (presumably the reported graphitized carbon) with comparably large caverns, disrupting the otherwise fluffy structure.

Compared to the cells with a Ni-fiber substrate on the cathode (Figure 1), the cells employing the carbon paper substrates reach much higher current densities (Figure 2d), in the case of the H23C2, even close to 3 A cm^–2^ at 2.3 V without any apparent mass transport limitation. The cell with the H24C5 reaches almost 2 A cm^–2^ but seems to encounter mass transport issues at above 1.5 A cm^–2^. The HFR (Figure 2d, bottom) decreases with higher current densities for both setups, except for the H24C5, where it increases slightly above the mentioned onset of limitation.

As the HFR at low current densities fits the expected ohmic drop across the separator very well (see eq 4), the decrease observed when increasing the current density must stem from a net increase in the ion mobility through the separator. The observed decrease of up to 50 mΩ cm^2^ would correlate to an increase in the specific conductivity of the electrolyte in the pores from 0.3554 S cm^–1^ ^10^ to roughly 0.5 S cm^–1^. If this effect was purely caused by higher local temperatures due to ohmic heating, a temperature within the separator of more than 100 °C would need to develop, which we deem unlikely. On the other hand, also, a local increase in OH^–^ concentration could lead to an increase in electrolyte conductivity, whereas an increase to 1.48 M KOH would need to be reached.^10^ While this value seems reasonable, a deeper discussion is not possible at the moment. Models dealing with the local hydroxide ion accumulation in the electrolyte for AWE have only been reported for industrially employed highly concentrated electrolytes and/or for the lower current densities commonly achieved.

While the results by Denk et al.^26^ could explain the elimination of the mass transport at lower current densities and could also explain the higher electrolyte conductivities within the separator at higher current densities – i.e., due to the more confined HER region close to the separator leading to a larger concentration gradient across – it can not explain the difference between the two carbon papers, as the catalyst layer brought onto their MPLs is identical in structure (see Figure 3).

**Figure 3:**
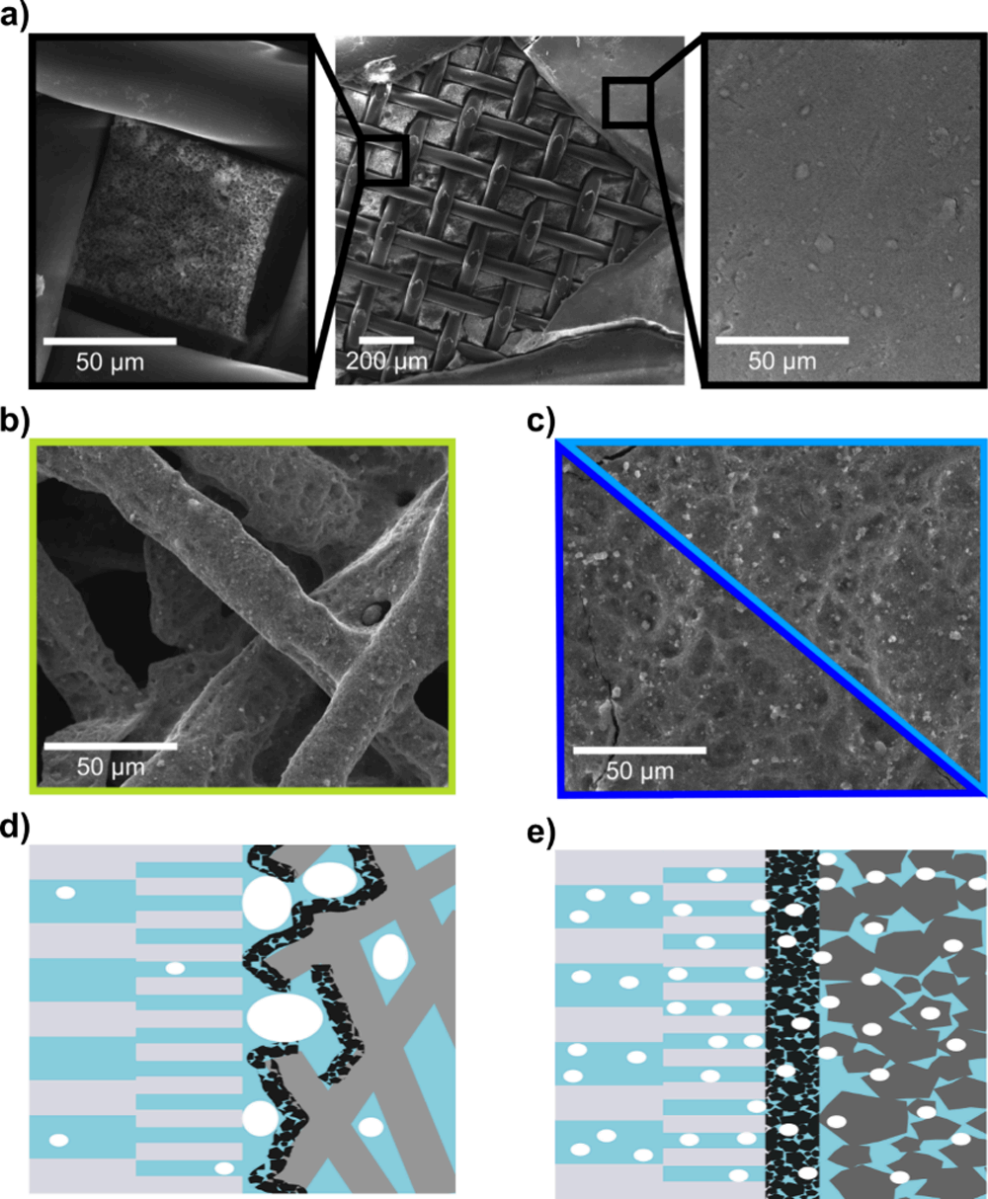
Top-view SEM pictures. (a) Overview of the Zirfon separator (center), where a part of the skin layer is removed by cryo-fracturing to reveal the bulk material with structural reinforcement (left) beneath the skin layer (right). (b) Cathode electrode with Ni-fiber as PTL substrate. (c) Cathode electrodes with carbon paper as substrate (H23C2: dark blue, H24C5: light blue). (d) Illustration of the Ni-fiber configuration, where accumulated bubbles do not penetrate the Zirfon skin layer. (e) Illustration of the carbon paper configuration, where small bubbles are formed directly at the CL/Zirfon interface, which causes them to infiltrate the Zirfon skin layer.

Figure 2d demonstrates that neither blocked active sites nor cell resistance alone is the cause for the increase in cell potential. The cell resistance and HFR corrected voltage (light blue hollow symbols) increase, where a strong mass transport influence is suspected, strengthening the theory of these two phenomena being strongly interconnected in this system.

Figure 2e shows the gas crossover for the two systems with carbon paper as cathode PTL. With between 30 and 40%_vol_ H_2_, these values are more than 10-fold of what was measured in the Ni-fiber case. Not only does this pose a safety risk when operated without additional flushing of N_2_, but this amount of hydrogen crossover will also drastically reduce the Faradaic efficiency of an electrolyzer, as almost half of the produced hydrogen is lost to the anode side.

The two carbon papers show no significant difference in gas crossover, signaling that the, e.g., wettability seems to play a minor role in this case. Due to the massive amount of hydrogen found in the anode exhaust, we do not expect this to be rationalizable by oversaturation effects of the lye.

### Necessary Oversaturation Factor for Carbon Paper PTL

In order to estimate the oversaturation factor ζ, a similar approach to Haug et al.^9^ was used. ζ represents the local concentration of dissolved hydrogen *c*_H_2__ compared to the equilibrium concentration according to Henry’s law.

$$\zeta =\frac{c_{H_{2}}}{p_{H_{2}}\cdot S_{H_{2}}}$$ 5

The driving force for the diffusive mass transport from cathode to anode is the local concentration difference at the diaphragm–electrode interface. We can therefore calculate the concentration difference based on the observed hydrogen crossover flux density *J*_H_2__^cross^ and the effective diffusion coefficient *D*_H_2__^eff^ by taking into account the structural parameters of the diaphragm τ and ε.^16^ The bulk diffusion coefficient of H_2_ in KOH at 70 °C ( $D_{H_{2}}^{1M\;\mathrm{KOH},70{\;}^{{}^{\circ}}C}\approx 1\times 10^{-8}\frac{m^{2}}{s}$ ) was estimated from the results of Tham et al.^30^

$$\left(c_{H_{2}}^{\mathrm{Cathode}}-c_{H_{2}}^{\mathrm{Anode}}\right)=J_{H_{2}}^{\mathrm{cross}}\cdot \frac{t}{D_{H_{2}}^{\mathrm{eff}}}=\frac{{ṅ}_{H_{2}}^{\mathrm{Anode}}\cdot t}{A\cdot D_{H_{2}}^{\mathrm{eff}}}=\frac{{ṅ}_{H_{2}}^{\mathrm{Anode}}\cdot t\cdot \tau }{A\cdot \varepsilon \cdot D_{H_{2}}^{1M\;\mathrm{KOH},70{\;}^{{}^{\circ}}C}}$$ 6

For small crossover fluxes, the concentration of dissolved hydrogen at the anode *c*_H_2__^Anode^ can commonly be neglected. Minding the high values measured for the carbon paper PTL case, this assumption will not be true. We therefore estimate the order of magnitude for the oversaturation factor using two extreme boundaries: (a) the oversaturation factor at the anode and cathode is the same, and (b) the oversaturation factor at the anode is 1.

$$\left(c_{H_{2}}^{\mathrm{Cathode}}-c_{H_{2}}^{\mathrm{Anode}}\right)=\zeta \cdot \left(p_{H_{2}}^{\mathrm{Cathode}}-p_{H_{2}}^{\mathrm{Anode}}\right)\cdot S_{H_{2}}^{1M\;\mathrm{KOH},70{\;}^{{}^{\circ}}C}$$ 7a

$$\left(c_{H_{2}}^{\mathrm{Cathode}}-c_{H_{2}}^{\mathrm{Anode}}\right)=\left(\zeta \cdot p_{H_{2}}^{\mathrm{Cathode}}-p_{H_{2}}^{\mathrm{Anode}}\right)\cdot S_{H_{2}}^{1M\;\mathrm{KOH},70{\;}^{{}^{\circ}}C}$$ 7b

The solubility of hydrogen in 1 M KOH at 70 °C was estimated based on the calculations by Haug et al.^9^ (which align well with the experimental results by Schalenbach et al.^31^) to be $S_{H_{2}}^{1M\;\mathrm{KOH},70{\;}^{{}^{\circ}}C}\approx 0.58\frac{\mathrm{mol}}{m^{3}\cdot \mathrm{bar}}$ , and the partial pressure of hydrogen at both electrodes was calculated assuming a water vapor pressure of roughly 0.3 bar and using the measured hydrogen share in the exhaust gas.

Inserting the indicated literature values, the measured hydrogen flux across the separator and the hydrogen share in the cell exhaust streams exemplarily for the measurement employing the H23C2 substrate leads to unrealistically high oversaturation factors ζ. The lower estimate assuming oversaturation to only occur at the gas evolving electrode (eq 7b) results in values of ζ_H_2__^0.5 A cm^–2^^ = 1193 and ζ_H_2__^1 A cm^–2^^ = 2186. The higher estimate assuming a constant oversaturation factor at the anode and cathode (eq 7a) even leads to values of ζ_H_2__^0.5 A cm^–2^^ = 1763 and ζ_H_2__^1 A cm^–2^^ = 3517.

Not only are these values much higher than expected to be realistic for oversaturation, but they also follow the current density, which deems a bulk movement of hydrogen bubbles through the porous separator the more likely explanation. Possible convective gas bubble movement through the separator (caused by the higher gas transport resistance through the carbon paper PTLs) might also decrease the ohmic resistance of the electrolyte. The addition of a convective force from cathode to anode – i.e., in the direction of the electro-osmotic drag – could enhance hydroxide ion movement to the anode.

With the substantial difference compared to the Ni-fiber case, it is likely that the overall structure of the used PTL substrate dictates the crossover when using a porous separator. To better understand the structural difference between the two types of PTL and how this can explain the performance difference as well as the crossover behavior, Figure 3 shows top view SEM pictures of the critical components.

### Unraveling the Structure–Performance Relationship in Zero-Gap Porous Separator Configurations

Figure 3a (center) shows an overview of the Zirfon separator. Part of the surface layer is fractured and removed by soaking the separator in liquid nitrogen and bending it afterward with tweezers. This reveals the bulk material with structural reinforcement (Figure 3a, left) beneath the skin layer (Figure 3a, right). The skin layer shows much smaller pores compared to the bulk material and is therewith meant to reduce gas crossover.^30,31^

The top view SEM picture of the Ni-fiber electrode (Figure 3b) reveals large gaps between the distinct fibers. The nanoporous CL is located on top of these fibers, so that only small parts of it are in direct contact with the Zirfon in an assembled cell. Figure 3c shows a continuous CL on top of the two respective carbon paper PTLs, which show no significant difference in macroscopic structure.

Figure 3d and e illustrates how the macroscopic structure of the used substrate in a zero-gap electrolyzer can influence the cell performance as well as the gas crossover if a porous diaphragm is used as a separator. In the case of the fiber sinter PTL (Figure 3d), the evolving gas bubbles can accumulate in the large pores between the fibers. This will render large portions of the catalyst disconnected ionically, as the bubbles will replace liquid electrolyte, and cause mass transport limitation, as was observed in Figure 1.

The bubble size is believed to be dictated mainly by the open pore structure of the PTL substrate. In the fiber case, the bubbles are then too large to penetrate the skin layer of the separator, which causes the comparably low measured gas crossover by size exclusion. Opposite to the fiber PTL case, the continuous microporous CL on the carbon paper substrates (Figure 3e) will prevent the accumulation of large bubbles, so that mass transport limitation is mitigated. At the same time, these microbubbles are believed to be small enough to pass through the skin layer of the separator along with the electro-osmotic drag, leading to concerning degrees of gas crossover. We expect this effect to be an inherent challenge when working with nanoparticle-based catalyst layers in combination with a porous separator. Dense layers as formed by catalyst coatings on the separator will enhance the electrical performance of the cell but at the cost of significant Faradaic efficiency losses and unsafe operation.

The fiber PTL and carbon paper substrate each have favorable properties, of which the combination is desirable for electrolyzers. The carbon paper configuration reaches high performances due to its advantageous mass transport behavior, but gas crossover needs to be inhibited. According to this hypothesis, one possibility to improve the carbon paper configuration would therefore be to hinder the gas bubbles to directly infiltrate the porous separator. While gas purity is no pressing issue for the Ni-fiber configuration, its performance suffers from mass transport limitation. The removal of accumulated gas bubbles must be improved to keep active sites from being blocked. Consequently, two more cell configurations, attempting these optimizations, were tested in this study.

### Introducing a Spacer between Electrode and Porous Separator

The first test was to modify the H23C2 configuration. Stainless steel meshes are commonly used to introduce a defined gap between the electrode and separator.^13^ In our study, a 150 μm stainless steel fiber sinter (similar in structure to the used Ni-fiber substrate) was introduced as a spacer between cathode and separator (Figure 4a). This is meant to increase the distance between CL and Zirfon and therefore reduce gas crossover while keeping the microporous continuous structure of the well performing CL.

**Figure 4:**
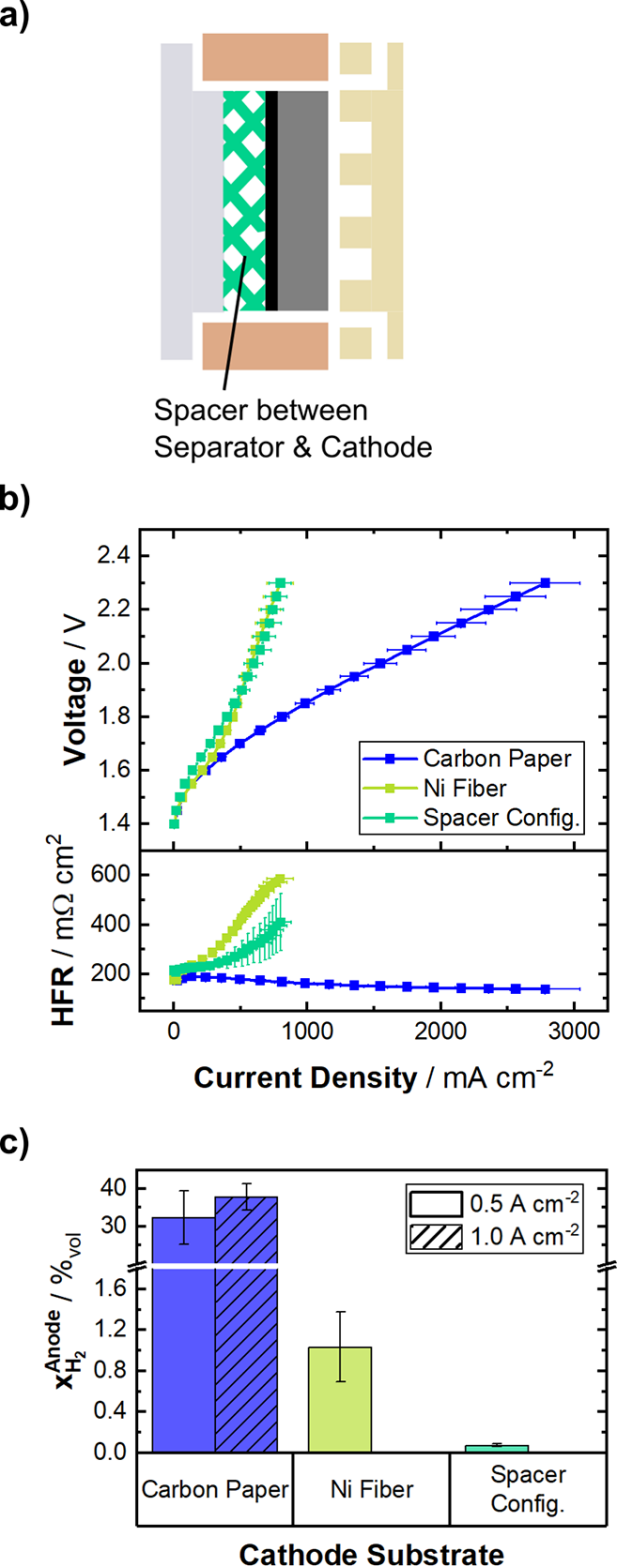
(a) Schematic of the cell configuration with a spacer between the electrode and separator. (b) Potentiostatic polarization curves (top) of the spacer configuration in comparison to the respectively best performing Ni-fiber (5%) and carbon paper (H23C2) configurations with their respective HFR (bottom) at 70 °C, symmetric 1 M KOH electrolyte feed and ambient pressure. (c) Hydrogen crossover, quantified as H_2_ in the anode gas stream at 500 mA cm^–2^ and 1 A cm^–2^. Data are depicted as mean values of three independent measurements with error-bars denoting the standard deviation.

The polarization curve (Figure 4b, top) and HFR (Figure 4b, bottom) of the spacer configuration (turquoise) show remarkably similar behavior to the cell, with the cathode catalyst being applied to the Ni-fiber PTL (green). As the stainless steel spacer has a similar open pore structure to the Ni-fiber PTL, bubble accumulation within the spacer is expected. The small gas bubbles produced within the nanoporous CL coalesce in the large pores provided by the spacer. This leads to comparable mass transport limitation in the spacer configuration as for the Ni-fiber cell. The high frequency cell resistance shows a similar increasing trend with current density at slightly lower values compared to the Ni-fiber configuration, even though the distance for ionic conduction should be elongated by use of the spacer. In the case of the spacer, the determination of the HFR could be affected by the use of stainless steel.

The HFR usually represents the combination of electrical resistance through periphery and PTL to an active site, where the charge transfer (i.e., reaction) happens and the resistance of ionic transport from the active site through the electrolyte to the other electrode. Stainless steel is in first approximation regarded as inert toward the reaction but still incorporates Ni, Mo, and other transition metals in its alloy, which can act as charge transfer sites^7,32^ in the high frequency impedance measurement. This could cause the HFR to depict the electrical resistance through the electrode and spacer, where the charge transfer happens at an “unintentional” active site, rather than the reaction at the “intended” site (i.e., the catalyst layer). An experiment with a truly inert and/or electrically nonconductive spacer would then allow the precise characterization of cell resistance but was out of scope of this study.

The gas crossover (Figure 4c) is reduced significantly by introducing a spacer, even compared with the Ni-fiber configuration. As mentioned above, the spacer is assumed to cause bubble accumulation similar to the Ni-fiber PTL, so that crossover is likely reduced by the same size exclusion mechanism. Additionally, the distance between the active site of the CL and the separator is increased, so that the majority of gas bubbles are kept from evolving directly into or close to the pores of the separator.

### Manipulating the Flow Pattern to Increase Mass Transport

The second test was to improve the gas removal from the electrode in the Ni-fiber PTL cell so that blocking of active sites would be prevented. The conventional flow pattern with exclusively diffusive mass transport within the PTL (Figure 5a, left) does not seem to be sufficient. KOH-resistant pieces were 3D-printed to block the flow channels of the flow field. By changing the cut-out of the gaskets, the liquid flux was forced through the Ni-fiber PTL (Figure 5a, right) in an attempt to effectively remove the bubbles from the electrode.

**Figure 5:**
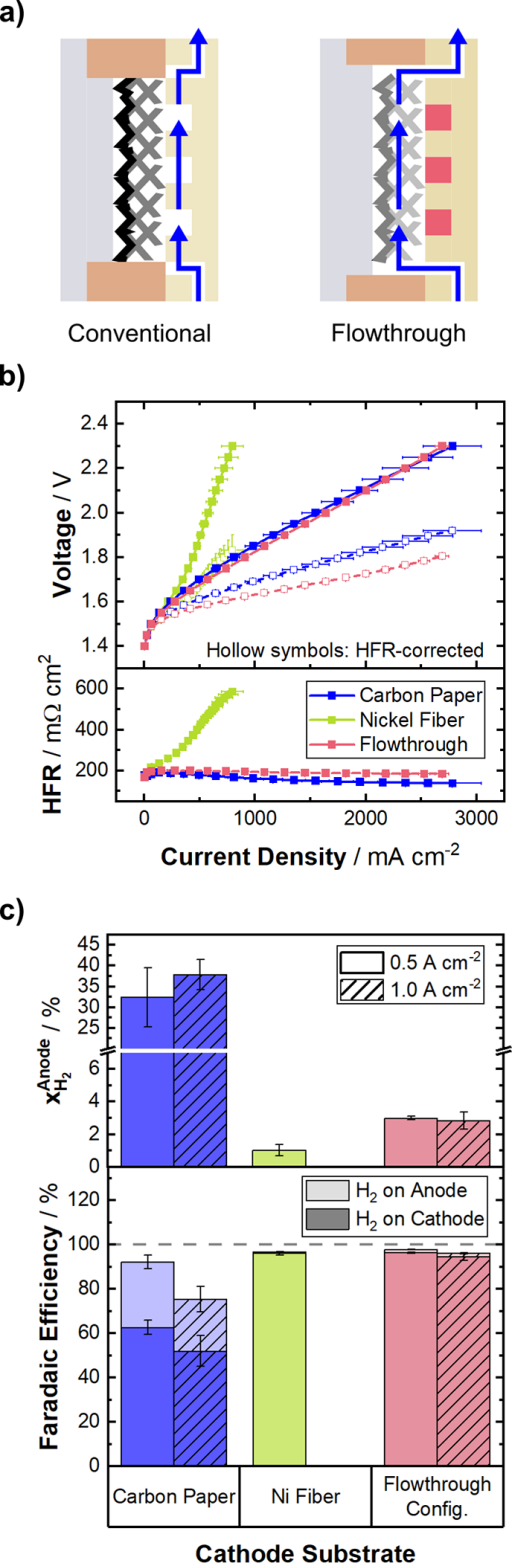
(a) Schematic of the regular flow pattern in a zero-gap cell (left) and the modified flow through the electrode (right). (b) Potentiostatic polarization curves (top) of the flowthrough configuration in comparison to the respectively best performing Ni-fiber (5%) and carbon paper (H23C2) configurations with their respective HFR (bottom) at 70 °C, symmetric 1 M KOH electrolyte feed, and ambient pressure. (c) Top: Hydrogen crossover, quantified as H_2_ in the anode gas stream at 500 mA cm^–2^ and 1 A cm^–2^. Data is depicted as a mean value of three independent measurements with error-bars denoting the standard deviation. Bottom: Faradaic efficiency at the cathode gas stream and loss of efficiency due to hydrogen crossover to the anode compartment.

When the electrochemical performance (Figure 5b) is compared with the conventional flow pattern (green), the new flowthrough configuration (red) shows a considerably improved performance with no sign of mass transport limitation. Compared to the carbon paper substrate (blue), the flowthrough electrode exhibits a more constant HFR throughout the current range, which results in an improved HFR-corrected polarization curve (hollow symbols).

The gas crossover (Figure 5c-top) is slightly higher for the flowthrough electrode, compared to the conventional flow pattern Ni-fiber cell, but with values below the important limit of 4%_vol_ H_2_. By changing the flow path of the electrolyte through the cathode substrate, the crossover could be reduced by 1 order of magnitude.

Figure 5c-bottom shows a breakdown of produced hydrogen in the cell exhausts. The Faradaic efficiency is calculated from the measured hydrogen flux in the cathode gas stream. The loss of efficiency resulting from H_2_ crossover is calculated from the measured H_2_ flux in the anode compartment and is stacked as semitransparent bars onto the Faradaic efficiency. The third part to close the hydrogen mass balance is likely to be lost to recombination with oxygen to form water, which was investigated in detail by Freiberg and Thiele.^22^ The carbon paper configuration shows significantly lower Faradaic efficiency than the two configurations employing the Ni-fiber as a substrate, which are both at similar levels.

The forced liquid flux through the electrode seems to alleviate the mass transport limitation by removing evolved bubbles effectively. Even though the carbon paper configuration did not show any obvious mass transport influence, the HFR-corrected polarization curve is improved for the flowthrough electrode. This correction leaves losses assigned to kinetics, mass transport, and parts of the electrode resistance not accounted for. As the catalyst material itself is unchanged (i.e., same kinetic losses are expected for constant catalyst utilization), this improvement can still be assigned to less bubble coverage in the CL, which improves catalyst utilization.

The hypothesis of the observed mass transport limitation to be actually caused by hydroxide ion depletion at the anode according to the results of Denk et al.^26^ cannot explain the results of the flow-through configuration, as the anode flow pattern was not changed. At most, the flow-through configuration should increase the mass transport limitation by limiting the local OH^–^ concentration increase at the cathode (and thereby reducing the driving force for the ion transport through the separator to the anode).

Both possible explanations for the decrease in HFR observed for the carbon paper PTLs (local concentration increase and bubble-enhanced ion movement through the separator) could be alleviated by the flow-through configuration. A deeper understanding of this behavior is currently under investigation.

As the gas crossover of the flowthrough electrode is within the same order of magnitude as the conventional Ni-fiber configuration, the Faradaic efficiency is also comparable between the two systems. The slight increase in crossover might stem from a pressure gradient as the blocked flow channels induce an increased backpressure in the cathode compartment. The resulting liquid drag makes the connection of the two electrolyte reservoirs via a liquid bridge mandatory for fill level equilibration. As the primary electrolyte reservoirs are still flushed with excess nitrogen, we do not expect significant gas impurities to be dragged along the liquid bridge.

In addition to being a major safety risk, gas crossover also drastically influences the efficiency of an electrolysis system and therefore its potential for industrial application. When the Faradaic efficiency of the flowthrough and Ni-fiber configuration is compared to the carbon paper cell (Figure 5c-bottom), the importance of gas crossover becomes imminent. Even though both systems (flowthrough Ni-fiber configuration and carbon paper at the cathode) reach similar current densities, the Faradaic efficiency of the carbon paper configuration is below 63%, rendering it unsuitable for industrial application.

### Transferability of Results for AWE

The motivation of this study was to show the possibility but also challenges when aiming for AWE cells to run at lower electrolyte concentration. Before the conclusion, we briefly discuss the placement of our results with respect to current achievements in AWE research and the transferability to traditional AWE operation employing highly concentrated electrolytes.

AWE cells do not rely on the use of scarce PGMs such as iridium and platinum as catalysts for the oxygen and hydrogen evolution reaction, which is currently seen as the biggest advantage in order to enable large-scale production of green hydrogen. Still, we employed PEMWE catalysts throughout this study due to their high chemical stability during short-term testing. Transition metal based catalysts change their structure during AWE operation. This will hinder the understanding of the dominating effect of the electrode structure on cell performance and crossover behavior. Iridium at the anode will need to be replaced by alkaline oxygen evolution catalysts, such as NiFe-LDH, which we are implementing currently. Preliminary data suggest that the effects presented here are directly transferable. Platinum for the alkaline hydrogen evolution reaction is more common in academia, although long-term stability is debated. Alternatives like Raney-nickel, however, tend to show strong structural changes over several hundred hours, which limits the direct transferability of our results. Additionally, the distribution of active sites will change significantly when going for a less active nanoparticle catalyst, with the substrate contributing to the gas evolution activity.

The two carbon paper based PTL materials presented in this study show the expected difference in cell polarization caused by their different electrolyte management, with the H23C2 exhibiting no mass transport limitation polarization characteristics. Still, the dense catalyst layer produced leads to an unacceptable crossover behavior. We assign this to be caused by the nanobubbles formed being able to penetrate the diaphragm. Without excessive flow engineering, such structures as formed on carbon paper based PTLs cannot be employed based on our current understanding. In our opinion, this is a key result that needs to be taken into consideration when employing nanoparticle based catalysts in the next generation of AWE. The catalyst layer structure formed out of the nanoparticles will significantly impact gas crossover behavior in zero-gap diaphragm based systems.

At last, the effect of electrolyte concentration has to be discussed. Commonly highly concentrated electrolyte solutions are used in AWE cells due to their superior conductivity and reduced gas solubility. However, such caustic conditions lead to the necessity of either working with polymer-lined balance of plant components (high CAPEX) or maintenance downtime with higher periodicity (high OPEX). By going for a zero-gap design with the new, thinner generation of diaphragms, it ought to be possible to go to lower lye concentrations. Despite the catalyst layer structure effect, also the awareness of the impact of electrolyte confinement upon gas evolution in substrates with smaller structural dimensions is transferrable to the operation of AWE cells with higher lye concentration, though the effect will be less pronounced, necessarily. The 1 M KOH used in this study was chosen intentionally. Based on literature, it is known that lower molarities eventually lead to hydroxide ion depletion at the anode, which does not just limit the maximum current density achieved but can also largely impact catalyst stability due to pH changes.

## Conclusions

In this study, we investigated zero-gap AWE cells with state-of-the-art nanostructured electrodes and moderate electrolyte concentration. Due to the restricted ionic conductivity of 1 M KOH, cells incorporating Ni-fiber PTLs as a cathode substrate are found to be prone to mass transport limitation and therefore bound to low current densities. This limitation can be alleviated but not completely solved by adjusting the cell compression within the active area, as this directly affects the porosity of the system. The remaining mass transport effect is ascribed to the accumulation of gas bubbles within the electrode. By using carbon paper as PTL material, the reached current density can be improved drastically, but gas crossover becomes an issue, as it reaches up to 40%_vol_ H_2_ in in the anode gas stream.

The analysis of the macroscopic structure of the involved components allows a structure–performance relationship to be formulated, where the gas bubble size is dictated by the open pore space in an electrode. The bubble size plays a crucial role, as it is assumed that large, accumulated gas bubbles in the fiber PTL case are blocked by the porous separator via size exclusion. Microbubbles in the case of the carbon paper PTL, on the other hand, are believed to have less effect on mass transport but are able to penetrate the separator and therefore cause high gas crossover. A potential solution is found by manipulating the flow path of the liquid electrolyte through the electrode, forcing convective flux rather than conventionally relying on diffusive transport. The resulting flowthrough electrode achieves >2.5 A cm^–2^ at 2.3 V without observable mass transport limitation and low gas crossover of <3%_vol_ H_2_ in the anode exhaust.

Even if the described consequences of electrode structure on performance are not as apparent in systems that employ more highly concentrated electrolytes and/or solid polymer electrolytes, the basic principles of bubble management are still believed to be applicable. Despite achieving membrane-like performance and gaining some valuable insight into the ongoing processes and relationships of this zero-gap system, it became evident that more fundamental understanding of effective bubble removal, compression, pressure operation, etc. is necessary to fully optimize such a system. We have also shown that optimization of the electrical performance of AWE cells must not be done without online gas analysis, as systems employing dense catalyst layers and other more complex electrode structures will face additional limitations with respect to gas crossover.

In addition to being a potential stand-alone electrolysis system, the zero-gap AWE cell offers the opportunity to characterize new catalyst­(-layer) materials and/or structures without the possibly overshadowing influence of state-of-the-art anion-exchange polymers at industry relevant current densities and overpotentials.

## †

Institute of Energy Technologies Elektrochemische Verfahrenstechnik (IET-4), Forschungszentrum Jülich, Wilhelm-Johnen-Straße, 52428, Germany (B.B.)

## ABBREVIATIONS

AWE: Alkaline Water Electrolysis
CAPEX: Capital Expenditure
CL: Catalyst Layer
CCS: Catalyst Coated Substrate
DI: Deionized
FIB-SEM: Focused Ion-Beam Scanning Electron Microscopy
HFR: High Frequency Resistance
IrOx: Iridium Oxide
MFC: Mass Flow Controller
MPL: Microporous Layer
MS: Mass Spectrometer
OPEX: operational expenditure
PEMWE: Proton Exchange Membrane Water Electrolysis
PGM: Platinum Group Metal
PTFE: Polytetrafluoroethylene
PTL: Porous Transport Layer
SEM: Scanning Electron Microscopy
SS: Stainless Steel
